# Supplementary material for: Pyruvate Dehydrogenase Kinase 1 inhibition mediated oxidative phosphorylation enhancement in cartilage promotes osteoarthritis progression
Source: BMC Musculoskelet Disord. 2023 Jul 20;24:597. doi: 10.1186/s12891-023-06585-6 (PMC10357736; doi:10.1186/s12891-023-06585-6)
Supplement: Supplementary file 1 — Additional file 1. [file 12891_2023_6585_MOESM1_ESM.zip › BP.Gsea.1653623667859/GOBP_RESPONSE_TO_GONADOTROPIN.html]

Details for gene set GOBP\_RESPONSE\_TO\_GONADOTROPIN[GSEA]

|  || Dataset | 55457\_collapsed\_to\_symbols.cls.cls #OA\_versus\_normal.cls.cls #OA\_versus\_normal\_repos |
| Phenotype | cls.cls#OA\_versus\_normal\_repos |
| Upregulated in class | normal |
| GeneSet | GOBP\_RESPONSE\_TO\_GONADOTROPIN |
| Enrichment Score (ES) | -0.5274274 |
| Normalized Enrichment Score (NES) | -1.7783418 |
| Nominal p-value | 0.0 |
| FDR q-value | 0.114982486 |
| FWER p-Value | 0.993 |
Table: GSEA Results Summary

  

Fig 1: Enrichment plot: GOBP\_RESPONSE\_TO\_GONADOTROPIN      
 Profile of the Running ES Score & Positions of GeneSet Members on the Rank Ordered List

  

| SYMBOL | TITLE | RANK IN GENE LIST | RANK METRIC SCORE | RUNNING ES | CORE ENRICHMENT || 1 | SRD5A1 | steroid 5 alpha-reductase 1 [Source:HGNC Symbol;Acc:HGNC:11284] | 1973 | 0.364 | -0.1071 | No |
| 2 | MAS1 | "MAS1 proto-oncogene, G protein-coupled receptor [Source:HGNC Symbol;Acc:HGNC:6899]" | 3629 | 0.191 | -0.2114 | No |
| 3 | PAX8 | paired box 8 [Source:HGNC Symbol;Acc:HGNC:8622] | 3680 | 0.187 | -0.1915 | No |
| 4 | EDNRA | endothelin receptor type A [Source:HGNC Symbol;Acc:HGNC:3179] | 4663 | 0.099 | -0.2552 | No |
| 5 | SLC5A5 | solute carrier family 5 member 5 [Source:HGNC Symbol;Acc:HGNC:11040] | 5151 | 0.060 | -0.2854 | No |
| 6 | WT1 | WT1 transcription factor [Source:HGNC Symbol;Acc:HGNC:12796] | 5452 | 0.037 | -0.3041 | No |
| 7 | GATA6 | GATA binding protein 6 [Source:HGNC Symbol;Acc:HGNC:4174] | 5537 | 0.030 | -0.3068 | No |
| 8 | LHCGR | luteinizing hormone/choriogonadotropin receptor [Source:HGNC Symbol;Acc:HGNC:6585] | 5679 | 0.017 | -0.3156 | No |
| 9 | FSHR | follicle stimulating hormone receptor [Source:HGNC Symbol;Acc:HGNC:3969] | 5757 | 0.011 | -0.3201 | No |
| 10 | CYP1B1 | cytochrome P450 family 1 subfamily B member 1 [Source:HGNC Symbol;Acc:HGNC:2597] | 5925 | 0.000 | -0.3331 | No |
| 11 | TGFBR3 | transforming growth factor beta receptor 3 [Source:HGNC Symbol;Acc:HGNC:11774] | 6868 | -0.069 | -0.3975 | No |
| 12 | EFNA5 | ephrin A5 [Source:HGNC Symbol;Acc:HGNC:3225] | 6997 | -0.080 | -0.3974 | No |
| 13 | PAPPA | pappalysin 1 [Source:HGNC Symbol;Acc:HGNC:8602] | 7867 | -0.146 | -0.4464 | No |
| 14 | CCNA2 | cyclin A2 [Source:HGNC Symbol;Acc:HGNC:1578] | 8047 | -0.159 | -0.4401 | No |
| 15 | GHSR | growth hormone secretagogue receptor [Source:HGNC Symbol;Acc:HGNC:4267] | 8472 | -0.191 | -0.4488 | No |
| 16 | GCLC | glutamate-cysteine ligase catalytic subunit [Source:HGNC Symbol;Acc:HGNC:4311] | 8534 | -0.196 | -0.4286 | No |
| 17 | PLAT | "plasminogen activator, tissue type [Source:HGNC Symbol;Acc:HGNC:9051]" | 8689 | -0.208 | -0.4141 | No |
| 18 | SRD5A2 | steroid 5 alpha-reductase 2 [Source:HGNC Symbol;Acc:HGNC:11285] | 10149 | -0.336 | -0.4848 | Yes |
| 19 | POR | cytochrome p450 oxidoreductase [Source:HGNC Symbol;Acc:HGNC:9208] | 10178 | -0.338 | -0.4440 | Yes |
| 20 | GCLM | glutamate-cysteine ligase modifier subunit [Source:HGNC Symbol;Acc:HGNC:4312] | 10439 | -0.366 | -0.4178 | Yes |
| 21 | GATA1 | GATA binding protein 1 [Source:HGNC Symbol;Acc:HGNC:4170] | 10958 | -0.425 | -0.4040 | Yes |
| 22 | INHBA | inhibin subunit beta A [Source:HGNC Symbol;Acc:HGNC:6066] | 11237 | -0.462 | -0.3670 | Yes |
| 23 | EDN1 | endothelin 1 [Source:HGNC Symbol;Acc:HGNC:3176] | 11276 | -0.468 | -0.3106 | Yes |
| 24 | NOTCH1 | notch receptor 1 [Source:HGNC Symbol;Acc:HGNC:7881] | 12555 | -0.776 | -0.3114 | Yes |
| 25 | NSMF | NMDA receptor synaptonuclear signaling and neuronal migration factor [Source:HGNC Symbol;Acc:HGNC:29843] | 12669 | -0.841 | -0.2134 | Yes |
| 26 | INHBB | inhibin subunit beta B [Source:HGNC Symbol;Acc:HGNC:6067] | 12680 | -0.851 | -0.1061 | Yes |
| 27 | MYC | "MYC proto-oncogene, bHLH transcription factor [Source:HGNC Symbol;Acc:HGNC:7553]" | 12801 | -0.970 | 0.0077 | Yes |
Table: GSEA details [plain text format]

  

Fig 2: GOBP\_RESPONSE\_TO\_GONADOTROPIN      
 Blue-Pink O' Gram in the Space of the Analyzed GeneSet

  

Fig 3: GOBP\_RESPONSE\_TO\_GONADOTROPIN: Random ES distribution      
 Gene set null distribution of ES for **GOBP\_RESPONSE\_TO\_GONADOTROPIN**
